# Lipid Droplets, Phospholipase A_2_, Arachidonic Acid, and Atherosclerosis

**DOI:** 10.3390/biomedicines9121891

**Published:** 2021-12-13

**Authors:** Miguel A. Bermúdez, María A. Balboa, Jesús Balsinde

**Affiliations:** 1 Instituto de Biología y Genética Molecular, Consejo Superior de Investigaciones Científicas (CSIC), 47003 Valladolid, Spain; mabermudez@ibgm.uva.es (M.A.B.); mbalboa@ibgm.uva.es (M.A.B.); 2 Centro de Investigación Biomédica en Red de Diabetes y Enfermedades Metabólicas Asociadas (CIBERDEM), 28029 Madrid, Spain

**Keywords:** lipid droplet, phospholipase A_2_, arachidonic acid, atherosclerosis, lipid signaling, lipid mediators, triacylglycerol

## Abstract

Lipid droplets, classically regarded as static storage organelles, are currently considered as dynamic structures involved in key processes of lipid metabolism, cellular homeostasis and signaling. Studies on the inflammatory state of atherosclerotic plaques suggest that circulating monocytes interact with products released by endothelial cells and may acquire a foamy phenotype before crossing the endothelial barrier and differentiating into macrophages. One such compound released in significant amounts into the bloodstream is arachidonic acid, the common precursor of eicosanoids, and a potent inducer of neutral lipid synthesis and lipid droplet formation in circulating monocytes. Members of the family of phospholipase A_2_, which hydrolyze the fatty acid present at the sn-2 position of phospholipids, have recently emerged as key controllers of lipid droplet homeostasis, regulating their formation and the availability of fatty acids for lipid mediator production. In this paper we discuss recent findings related to lipid droplet dynamics in immune cells and the ways these organelles are involved in regulating arachidonic acid availability and metabolism in the context of atherosclerosis.

## 1. Lipid Droplet Biogenesis. General Aspects

Mammalian cells accumulate excess neutral lipids, i.e., triacylglycerol (TG) and cholesterol esters (CE), in cytoplasmic organelles known as lipid droplets (LD). While LDs are present in almost all known cell types, their number, size and composition vary greatly from cell to cell and even within a given cell type. LDs are spherical particles composed of a phospholipid monolayer which encases a core made up mainly of TG and CE [1,2,3]. A large number of unique phospholipid molecular species has been identified in the LD monolayer. Most of these belong to the choline- and ethanolamine-containing glycerophospholipid classes (abbreviated as PC and PE, respectively), which represent approx. 64% and 24% of the total. Minor amounts of other classes such as phosphatidylinositol (PI), phosphatidylserine (PS), phosphatidic acid (PA), sphingomyelin, and lysophospholipids have also been identified [1,2,3].

The phospholipid monolayer is decorated with a variety of proteins that play numerous roles, ranging from regulating lipid mobilization from and to the organelle, to serving to support and stabilize the organelle structure itself. Of particular importance is the PAT family of proteins, which includes perilipin-1 (PLIN1), adipophilin (ADPR, PLIN2) and TIP47 (Tail-Interacting protein of 47 Kda, PLIN3) [4]. PLIN1 contributes to the growth and stabilization of LDs [5]. PLIN2 is involved in adipocyte differentiation and appears to be important for proper hydrolysis of the LDs [6]. The functions of PLIN3 are related to the regulation of lipolysis in skeletal muscle cells [7]. PLIN4 has been implicated in stabilizing the LD by interacting directly with TG moieties when their phospholipid coverage is limited [8]. PLIN5 appears to be involved in protective roles against mitochondrial damage and endoplasmic reticulum stress in liver [9].

In addition to the PAT proteins, numerous enzymes related to lipid metabolism have also been found on the surface of LDs; these include adipose triacylglycerol lipase (ATGL) [10], hormone-sensitive lipase [11], cytidine triphosphate:phosphocholine cytidylyltransferase [12,13], lysophosphatidylcholine acyltransferases [14], long-chain acyl-CoA synthetase 3 [15], lipin-1 [16,17], cyclooxygenase [18], and group IVA cytosolic phospholipase A_2_ (cPLA_2_α) [19,20], among others. Some of these proteins also participate in signaling [1,21,22,23,24]. Thus, the presence of such a varied number of lipid-metabolizing enzymes attests to the role of LDs as intracellular hubs where lipid signaling enzymes dock and interact to activate select pathways [1,21,22,23,24].

**Figure 1:**
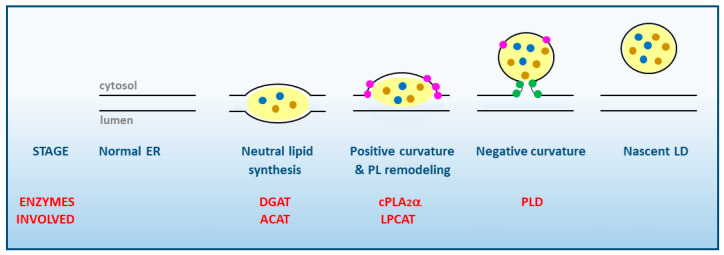
Stages of LD synthesis at the cytosolic face of smooth ER membranes. The various enzymes of lipid metabolism participating in each stage are highlighted in red. Note the essential role of cPLA_2_α in favoring the induction of positive membrane curvature. TG and CE inside the LD are represented by brown and blue dots, respectively. Lysophospholipids at the membrane are highlighted as pink dots, phosphatidic acid as green dots. PL, phospholipid; DGAT, diacylglycerol:acyl-CoA acyl transferase; ACAT, acyl-CoA:cholesterol acyl transferase; PLD, phospholipase D; LPCAT, lysophosphatidylcholine:acyl-CoA acyl transferase.

Although significant progress has been made in recent years, the mechanism of LD biogenesis is yet to be fully established. Formation of LDs involves a large number of steps and is carried out in the endoplasmic reticulum (ER), where the amphipathic monolayer of LDs originates [25,26] (Figure 1).

The first step in LD formation is the synthesis of TG and CE, the major neutral lipids that compose the core of the LD. Different enzymes located in the ER are involved in their synthesis, namely diacylglycerol acyltransferases (DGAT) for TG, and acyl-CoA:cholesterol acyltransferases (ACAT) for CE. These enzymes may be activated by different stimuli including excess free fatty acids present in the medium, cell activation via surface receptors, or ER stress [27]. The free fatty acids that are used for neutral lipid synthesis may arise from multiple sources, both exogenous (lipoproteins from blood plasma), and endogenous (destruction of intracellular membranes or stimulation of the de novo fatty acid biosynthesis) [27].

A widely held assumption to explain the formation of LDs posits that the newly formed TG and CE accumulate in the ER. Some studies demonstrated that up to 3 mol% of TG and 5 mol% of CE can be accommodated before the LD breaks off to the cytosol, while other studies have suggested even greater accumulation, nearing 5–10% of both TG and CE. In either case, a highly regulated rate of phospholipid to neutral lipid must be maintained at all instances to support the shape and biophysical properties of the nascent organelle [28,29].

In order to grow, nascent LDs need a positive local membrane curvature while still in the ER membrane (Figure 1). This is achieved by the accumulation of lysophospholipids particularly lysoPA, lysoPC and lysoPE which, having a wedge-shaped conformation, help the LD to grow and counteract the neutral or slightly negative curvature produced by PC and PE, respectively [3,25,28,29]. Since lysophospholipids are generated by phospholipase A_2_s (PLA_2_), this kind of enzymes, in particular cPLA_2_α, are key regulators of the initial stages of LD formation [24,30,31,32,33]. Of note, cPLA_2_α manifests a marked selectivity for hydrolyzing phospholipids containing arachidonic acid at the sn-2 position [34], which makes the activation of this enzyme a key regulatory event for the synthesis of arachidonate-derived bioactive eicosanoids [35,36,37]. LDs have repeatedly been suggested to constitute a prominent eicosanoid-synthesizing site within the cells [38,39]; thus, it appears that cPLA_2_α may simultaneously serve two key functions of LD biology, i.e., to provide lysophospholipids to allow for continued growth of the organelle and, at the same time, to provide free AA substrate for the synthesis of bioactive lipid mediators in situ. This is further discussed in Section 7 below.

Once a positive curve has been generated at the LD baseline, cells are now able to store more TG and CE in between the phospholipid monolayers. As a result of such enhanced accumulation of neutral lipids, a simultaneous increase in the amount of PLs is necessary to maintain the biophysical properties of the monolayer of the nascent LD. This process requires the coordinate action of enzymes from the phospholipid biosynthetic and acyl chain remodeling pathways (i.e., phospholipid:acyl-CoA acyltransferases), the latter of which contribute to remove the lysophospholipids that accumulated in the membrane as a result of PLA_2_ action. As the LD emerges from the ER, a neutral curvature is established that stabilizes and protects the hydrophobic core from lipolysis [3,12,29].

In the latter stages of LD biogenesis, a negative curvature is generated at the connection sites with the ER to allow for the complete cleavage and release of the newly-formed LD to the cytosol. This effect is made possible by the accumulated presence of PA at the connection sites, which promotes a strong negative curvature [3,40]. PA is thought to be produced by phospholipase D enzymes [41,42] and removed, as needed, by lipin-1 [16,17]. Along the whole process described above, recruitment of a wide range of accessory proteins to the LD continuously takes place, such as the aforementioned proteins of the PAT family, which help stabilize the structure and assist in the recruitment of additional proteins and lipids [1,3,28,29,43].

Within the frame of the LD biogenesis mode depicted in Figure 1, many important questions still remain controversial and await for a definitive response [1,3,28,29]. For example, the specific sites of formation of neutral lipids, and growth of the LD within the ER remain questioned [3,28,29,44]. It is also largely unknown which of the LD-associated proteins are essential for the formation of the organelle, and how and why only some of these nascent LDs are selected for further expansion [45,46].

## 2. Functional Diversity of Lipid Droplets

From being considered merely as cellular storage depots of excess fatty acids destined for β-oxidation and membrane biosynthesis, LDs are now recognized as key players in cellular regulation and signaling, extra- and intracellular trafficking, and transcriptional gene regulation [1,3,28,29]. In addition, LD formation is also regarded as an indicator of stress and cell death [47]. LDs are also relevant to innate immunity and inflammation reactions, as their composition changes in response to danger signals by reprogramming cellular metabolism and activating antimicrobial mechanisms [48,49]. LDs facilitate the early antiviral immune response specifically through the enhanced modulation of interferon signaling that follows viral infection, and the control of viral replication [50].

Given the variety of functions associated to LDs, it is no surprise that LDs from various cell types, or generated under different stimulatory or metabolic conditions, may differ in their protein and/or lipid compositions. Different LD populations within the same cells may co-exist; in adipocytes, nascent LDs present mostly PLIN3 and PLIN4, while PLIN1 and PLIN2 are the predominant proteins in “older” LDs [51]. LD heterogeneity is also evident with regard to their lipid composition [52]. The use of Raman scattering microscopy has revealed that LDs present in the same cell may also sport different lipid compositions [53]. These findings have suggested that LDs may undergo maturation processes.

Proteomic studies are allowing the identification of an increasing number of proteins that differ among various LD populations [54]. Full characterization of the effect of these proteins on the biology of LDs will provide a better explanation of their heterogeneity and help clarify the interactions of LDs with the ER, the mitochondria, the peroxisomes, the endosomes, the lysosomes, and the plasma membrane [29,55]. In this regard, recent studies have identified a subpopulation of mitochondria adhered to LDs called peridroplet mitochondria, which appear to exhibit distinct bioenergetics, proteomics and dynamics that influence LD accumulation. Understanding the functions and regulation of mitochondrial attachment to LDs offers new points of intervention in metabolic diseases [56].

## 3. Lipid Droplets and Atherosclerosis

Imbalance of LD function has been implicated in a number of human pathologies. Overabundant and enlarged LDs are the hallmark of obesity, type 2 diabetes mellitus, hepatic steatosis, atherosclerosis, cardiac steatosis and cardiomyopathy [1]. While much effort has been devoted to characterizing LDs and their contribution to adipose tissue and liver pathobiology, lesser attention has been given to the role of LDs in cardiovascular disease [57]. A remarkable example of the importance of LDs to cardiovascular disease is the accumulation of CE and TG in these organelles in fat-laden macrophages (foam cells) in the artery walls, a key step for the development of atherosclerosis. However, the composition of LDs in the foam cells and the manner how LD formation and mobilization are regulated in the context of atherosclerosis are still poorly defined [57]. Atherosclerosis is the main pathology behind cardiovascular disease, and the leading cause of death in developed countries [58]. It can evolve asymptomatically for decades, consisting primarily of a thickening of the intima, the innermost layer of blood vessels, through the deposition of lipids, cells and extracellular matrix. This leads to the formation of the so-called atheroma plaques, which cause narrowing and loss of elasticity of blood vessels. Over time, some of these lesions can necrotize and calcify, leading to plaque rupture and thrombus release, increasing the risk of heart attack, stroke, or sudden cardiac death.

**Figure 2:**
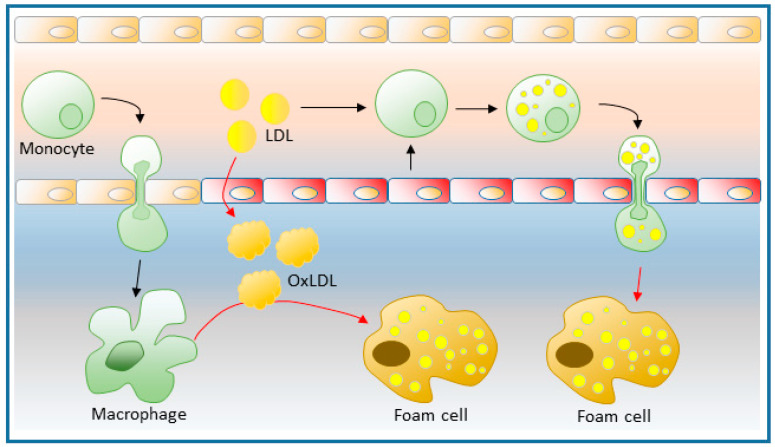
Role of monocytes and macrophages in the initiation of atherosclerotic plaque formation. Different factors leading to endothelial damage promote increased permeability to LDLs and facilitate the entry of monocytes into the subendothelial space, where they differentiate into macrophages. These increase the expression of scavenger receptors, which are responsible for endocyting oxidized LDL, and turning into foamy macrophages, which will be the main cause of the development of atherogenic plaques. In a state of dyslipidemia, circulating monocytes may form LD before migrating to the subendothelial space. This process may occur by the uptake of excess LDL, and also by interaction with products released by activated endothelial cells. The formation of foamy monocytes is accompanied by their inflammatory activation, which makes them more prone to subendothelial migration and thus more atherogenic.

Several studies on the earliest stages of atherogenesis in humans and animal models have shown that the key initial step is the subendothelial retention of apolipoprotein B-containing lipoproteins (apoB-LPs) [59]. ApoB-LPs, which are produced by the liver and the intestinal cells, consist of a nucleus composed of TG and CE surrounded by a monolayer of phospholipids and proteins, similar to the LDs. Hepatic apoB-LPs are secreted as very low-density lipoproteins, which are converted in circulation into low density lipoproteins (LDLs). This accumulation of LDLs in the subendothelium will produce an inflammatory response, which is enhanced when the LDLs become oxidized. Activated endothelial cells become more permeable to the extravasation of lipoproteins, express adhesion molecules such as VCAM-1 and ICAM-1, secrete chemokines such as monocyte-chemoattractant protein-1 (MCP-1) that attract circulating monocytes, expose adhesion receptors such as P-selectins in the surface, and release pro-inflammatory cytokines, eicosanoids and large amounts of unmetabolized free AA [60,61,62,63,64] (Figure 2).

Most monocytes that have gained access to the subendothelial space in early atherosclerotic lesions become cells with macrophage and/or dendritic cell characteristics, favored by the increased presence in situ of multiple differentiation factors secreted by the endothelial cells [65,66,67]. By internalizing modified LDL and storing its lipid constituents into cytosolic LDs, monocytes become fat-laden macrophages or foam cells, which reside in the vessel wall. As a consequence, a systemic low-grade inflammation is chronically established. Recent work has shown that a constant flux of monocytes from the bloodstream into the intima appears to be necessary for the atheromatous plaque to effectively progress [68]. Interest in the heterogeneity of macrophages in atherosclerotic lesions, in particular with regard to the macrophages involved in a proinflammatory process versus those participating in resolution and repair events, has prompted a large number of recent studies [69,70]. However, the results are yet inconclusive because in vitro studies do not adequately reproduce the situation existing in the atherosclerotic endothelium. The latter involves many different cell types, the role of which can vary according to the stage of the disease. For the purposes of this review, emphasis is placed on the role of circulating monocytes and the macrophage cells into which they differentiate. As pointed out above, the formation of LDs in the cytosol of these cells is a key factor to be taken into account in the onset of the disease.

## 4. Circulating Foamy Monocytes

The fact that circulating monocytes are attracted to damaged areas of the endothelium and, once inside the intima, turn into foamy macrophages, is a concept that has been firmly established for decades [61,69,70]. Nevertheless, another model has emerged according to which monocytes interacting with products released by endothelial cells may acquire a foamy phenotype long before crossing the arterial endothelial barrier and differentiating into foamy macrophages in the intima [71,72,73,74,75] (Figure 2). In support of this concept, circulating leukocytes from patients with atherosclerosis in the coronary arteries have been shown to accumulate TG and CE [76]. Circulating leukocytes from patients with hypercholesterolemia also contained high amounts of LDs [77]. More recent data have shown that human peripheral blood monocytes increase their LD content after a high fat intake compared to levels found in monocytes from fasting individuals, whose LD levels are very low [78,79]. It is suggested that the increased formation of LDs under these conditions is due to the uptake of free fatty acids arising from the action of endothelial cell surface lipases on the TG transported by very low-density lipoproteins [79,80,81,82]. Similarly, mouse models prone to develop atherosclerotic plaques spontaneously such as apoE−/− or LdLr−/−, characterized by high circulating LDL, show an exacerbated LD content in their monocytes after one week on a high-fat diet; when fed a normal diet, they still exhibit more LD than wild-type mice [74,83].

Further, it has been reported that the synthesis of LDs from circulating foamy monocytes is accompanied by the increased expression of a number of surface integrins such as VLA-4, CD11b, CD11c, CD18 and CD29, and also chemokine receptors such as CCR2 and CX3CR1 [71,72,84,85]. This increased expression of integrins and receptors facilitates the adhesion of the foamy monocytes to endothelial cells, leading to their activation, and subsequent production of a number of cytokines, including IL-6, TNF-α, IL-8, IL-1β and IFN-γ [71,72,73,74,84,85]. Thus, accumulation of LDs in monocytes accompanies other phenotypic changes of the cells that increase the risk of cardiovascular disease. Therefore, circulating foamy monocytes could be considered not only as a potential therapeutic target but also as a marker for early atherosclerosis. Confirmation of a direct relationship in vivo between the amount of monocyte LDs and early development of atherosclerotic lesions was shown when circulating foamy cells (CD11c+) were eliminated with clodronate from high-fat diet-treated mice. This resulted in a significant reduction of atherosclerotic lesions [74].

## 5. Arachidonic Acid, a Compound Released in Atherosclerotic Lesions

Arachidonic acid (5,8,11,14-eicosatetraenoic acid, 20:4n-6, AA) is a member of the n-6 family of polyunsaturated fatty acids and can be obtained directly from the diet or synthesized from linoleic acid (18:2n-6) through the successive actions of Δ6-desaturase, elongase and Δ5-desaturase. Although produced mainly in the liver, practically all cells in the body are endowed with the machinery to produce AA from linoleic acid [86].

**Figure 3:**
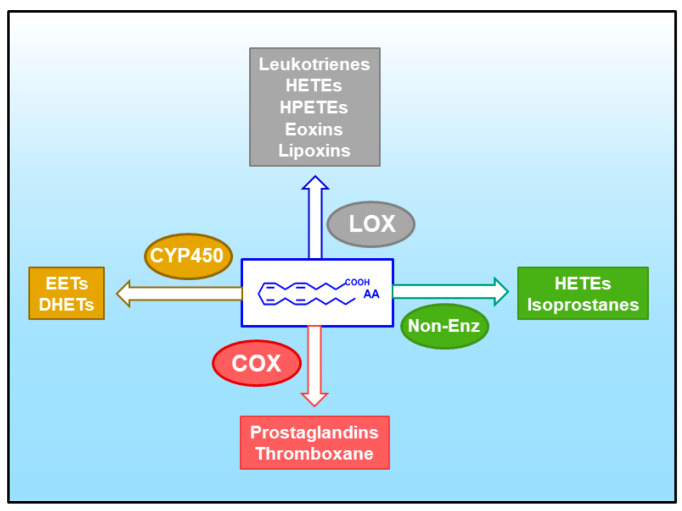
Pathways for the oxidative metabolism of AA. A variety of eicosanoids can be produced through four major pathways. These are: (i) the cyclooxygenase (COX) pathway, yielding prostaglandins and thromboxane; (ii) the lipoxygenase (LOX) pathway, yielding leukotrienes, hydroxyeicosatetraenoic acids (HETEs), hydroperoxyeicosatetraenoic acids (HPETEs), eoxins and leukotrienes; (iii) the cytochrome P450 (CYP450) pathway, yielding epoxyeicosatrienoic acids (EETs) and dihydroxyeicosatrienoic acids (DHETEs); and (iv) non-enzymatic oxidation reactions (Non-Enz), yielding isoprostanes and HETEs.

AA is the common precursor of the eicosanoids, a family of lipid mediators with key roles in physiology and especially in pathophysiological situations involving inflammatory reactions [36] (Figure 3). The potent biological activity of the eicosanoids requires the cells to exert a tight control on AA levels in a way that the availability of the fatty acid in free form is often a limiting factor for eicosanoid biosynthesis [87,88]. Of note, when present at sufficiently high concentrations, AA may be significantly converted to its two-carbon elongation product, adrenic acid (7,10,13,16-docosatetraenoic acid, 22:4n-6) [89,90,91], which can also be oxygenated to a variety of bioactive products [92,93,94].

**Figure 4:**
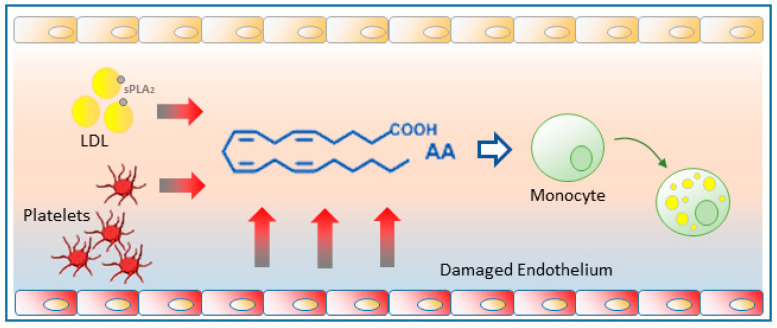
Free AA production in atherosclerotic foci. Damaged endothelium releases substantial amounts of free AA which can be taken up by circulating monocytes and promote their change to a foamy phenotype. Platelets recruited to the activated endothelium also contribute to generating free AA. The sPLA_2_ action on circulating LDLs can also provide significant amounts of free AA.

It has long been recognized that free AA is liberated in significant amounts into the bloodstream during the early stages of atherosclerosis [95]. Endothelial cells activated by oxidized LDLs and other stimuli constitute a major source of free AA [96,97,98]. In addition, the action of sPLA_2_s released from a variety of cells directly on the LDL-bound phospholipids constitutes another important source of AA released into the bloodstream [99,100,101,102]. Finally, platelets recruited to the activated endothelium also contribute to increase the availability of free AA [95] (Figure 4).

Increased levels of free AA in the vicinity of atherosclerotic lesions may contribute greatly to LD formation by circulating monocytes, thereby transforming them into foamy cells and hence, into pro-atherogenic monocytes. This view has been experimentally supported by data demonstrating that free AA, at pathophysiologically relevant concentrations [103], is a strong inducer of LD formation in human peripheral blood monocytes [30]. Free AA appears to serve two different roles; on the one hand it serves as a substrate for the synthesis of TG but, interestingly, not CE. Conversely, AA activates intracellular signaling via p38 and JNK-mediated phosphorylation cascades that enhance neutral lipid synthesis and also activate cPLA_2_α, which in turn is required to regulate the biogenesis of the LD [30].

LD production by monocytes exposed to AA proceeds the same in the presence of a number of cyclooxygenase or lipoxygenase inhibitors [30]. Moreover, removal of the oxidized impurities from the commercial fatty acid (hydroxy, hydroperoxy and oxo derivatives of AA generated spontaneously) used in the above studies yielded a fraction of pure free AA that completely recapitulated LD formation in monocytes, while the oxidized AA fraction did not [104]. These results demonstrate that it is free AA itself and not an oxygenated product that is responsible for inducing the conversion of monocytes into foamy cells.

Molecular analyses of the lipid composition of the LDs produced by AA in foamy monocytes has shown that the neutral lipid fractions of these cells are enriched with an uncommon positional isomer of palmitoleic acid, namely cis-7-hexadecenoic acid, (16:1n-9) [75]. 16:1n-9 exhibits significant anti-inflammatory activity both in vivo and vitro which is comparable to that of n-3 fatty acids, and clearly distinguishable from that of palmitoleic acid [75,105]. Further, 16:1n-9 can be mobilized from phospholipids in activated phagocytic cells to form novel lipid species such as 16:1n-9-containing PI molecules and esters of 16:1n-9 with various hydroxyfatty acids [106]. These two kinds of compounds have been shown to possess growth-factor-like properties [107] and anti-diabetic/anti-inflammatory activities [108], respectively. The selective accumulation in the neutral lipid fraction of phagocytic cells of an uncommon fatty acid such as 16:1n-9 may reveal an early phenotypic change that could provide a biomarker of proatherogenicity, and a potential target for pharmacological intervention in the first stages of cardiovascular disease. Intriguingly, the 16:1n-9 fatty acid has also been recently identified in the TG fraction of some cancer cells [109], suggesting perhaps a wider biological role.

## 6. Glycerophospholipid Hydrolysis as a Major Pathway for the Mobilization of AA

The production of lipid mediators is intrinsically linked to the availability of polyunsaturated fatty acid (PUFA) precursors necessary for their synthesis. This depends on the activities of numerous enzymes and proteins that regulate the uptake, transport, storage, hydrolysis, remodeling and trafficking of PUFA among the different cellular and extracellular lipid pools. The major metabolic route supplying free PUFA for lipid mediator synthesis is that regulated by PLA_2_s, because these enzymes can directly access the major cellular reservoir of readily mobilizable fatty acid, i.e., the sn-2 position of membrane glycerophospholipids, mainly PC, PE, and PI [35,36,37]. It is relevant to indicate, however, that despite the overwhelming quantitative importance of PLA_2_s to overall PUFA release, there are other minor routes not involving PLA_2_ which can play important roles under limited, tightly controlled conditions. These include monoacylglycerol lipases acting on endocannabinoids [110,111], acid lipases acting on lysosomal lipids [112], and triacylglycerol lipase (ATGL) acting on TG [113,114].

The PLA_2_ enzymes typically involved in cellular signaling leading to lipid mediator production have been classically categorized into three major families, namely the Ca^2+^-dependent cytosolic enzymes (cPLA_2_), the Ca^2+^-independent enzymes (iPLA_2_), and the secreted enzymes (sPLA_2_). A number of excellent comprehensive reviews covering the classification, characteristics and activation properties of the more than 30 members of the PLA_2_ superfamily have recently been published, and the reader is kindly referred to these for specific details [34,115,116,117,118,119,120,121,122,123,124,125].

The group IVA PLA_2_, or cPLA_2_α, is widely recognized as the key enzyme effecting the AA release because of its unique preference for AA-containing phospholipid substrates and its activation properties, which place it at the center of a number of key signaling pathways involving phosphorylation cascades and/or intracellular Ca^2+^ movements [34,117,118]. In accordance, studies using cPLA_2_α-deficient mice have confirmed that this enzyme is essential for stimulus-induced eicosanoid production in practically all cells and tissues [34,115,116,117,118]. A myriad of stimuli, acting on surface receptors, are able to trigger the translocation activation of cPLA_2_α from the cytosol to a number of intracellular membranes, including the LD monolayer [19,20]. This allows positioning of the enzyme in the vicinity of cyclooxygenases and lipoxygenases for efficient supply of the free fatty acid for eicosanoid formation [126,127].

The group VIA calcium-independent PLA_2_, frequently referred to as iPLA_2_β, is another important enzyme for lipid mediator production which, unlike cPLA_2_α, does not manifest overt specificity for any particular fatty acid, being able to efficiently hydrolyze all kinds of phospholipid substrates [128]. Studies in macrophages have suggested that cPLA_2_α and iPLA_2_β preferentially act on different membrane phospholipid subsets, the former cleaving AA-containing phospholipids, and the latter liberating other fatty acids, such as adrenic acid and palmitoleic acid [89,90,129]. These in vivo preferences suggest that the activity of each PLA_2_ in stimulated cells can also be limited by the nature of the stimulus, the subcellular localization of the enzyme, and the accessibility to a given phospholipid pool [130,131].

The secreted PLA_2_s (sPLA_2_) constitute the third family of PLA_2_ enzymes involved in lipid mediator production [120,132]. The sPLA_2_s are secreted by a variety of cells, particularly those of the innate immune system, and act mainly on extracellular substrates such as lipoproteins, microparticles, bacteria and viruses, and the outer plasma membrane of mammalian cells. In some cases, they can also be incorporated back to the cells that first released them or to neighboring cells, and act on different intracellular membrane locations to regulate innate immune responses [133,134,135]. sPLA_2_ enzymes often act in concert with cPLA_2_α to assist and/o amplify the cPLA_2_α regulated response leading to PUFA mobilization [136,137,138]. However, under some circumstances, some sPLA_2_s, especially those of groups IID, III, and X, elicit significant production of PUFAs and associated mediators by acting on their own on different extracellular targets, and showing certain degree of selectivity for hydrolysis of PUFA over other monounsaturated/saturated fatty acids [139,140,141,142].

While less generally recognized, free AA levels can also be significantly increased in cells if the reacylation of lysophospholipids is inhibited [37,87,118,143,144,145]. This is because AA is an intermediate of a reacylation/deacylation cycle, the so-called Lands cycle, where the fatty acid is hydrolyzed from phospholipids by PLA_2_s and reincorporated back by CoA-dependent acyltrasferases [37,87]. In resting cells, reacylation reactions dominate and, as a result, free AA levels in unstimulated cells are kept at very low levels. In stimulated cells, activation of cPLA_2_α makes deacylation to dominate over reacylation, which results in the net accumulation of free AA. Nevertheless, AA reacylation under activation conditions is still significant, as demonstrated by the finding that a large amount of the AA initially liberated by cPLA_2_α is returned back to phospholipids. Hence, blockade of the CoA-dependent acyltransferases involved in phospholipid AA reacylation can result in greatly elevated levels of the free AA which is then available for eicosanoid synthesis [87]. The substrate specificity of the acyltransferases involved in AA reacylation is the reason why this fatty acid is overwhelmingly incorporated in the position sn-2 of phospholipids [118].

A third layer of complexity in the regulation of free AA availability stems from the fact that, once incorporated into phospholipids, the AA does not remain in the phospholipid molecular species that initially incorporated it but moves between different phospholipid species by a series of CoA-independent transacylation reactions [37,145,146]. These reactions are key for the cells to maintain the appropriate distribution of AA within the various cellular pools so that, depending on stimulation conditions, they are accessible to the relevant PLA_2_ [130,131]. This is an important aspect for the regulation of eicosanoid synthesis, as the quantity and distribution of eicosanoids produced under a given condition also depends on the composition and cellular localization of the phospholipid pool where the AA-hydrolyzing PLA_2_ primarily acts [130,131].

These transacylation reactions are catalyzed by CoA-independent transacylase (CoA-IT), an enzyme that primarily transfers AA moieties from PC (diacyl species) directly to PE (both diacyl and plasmalogens species), circumventing the need for CoA or ATP [37,145,146]. The sequence of CoA-IT is still unknown which has made it difficult to advance our knowledge on the cellular regulation of phospholipid transacylation. Recent studies have provided suggestive evidence that the CoA-IT-mediated reaction is primarily catalyzed by a well described PLA_2_ enzyme, the group IVC phospholipase A_2_γ (cPLA_2_γ) [130,146]. Unlike its homolog cPLA_2_α, cPLA_2_γ is a calcium-independent enzyme and does not manifest clear selectivity for AA residues [147]. Since the CoA-IT reaction involving AA-containing phospholipids appears to be critically involved in the inflammatory response of macrophages to certain stimuli [131,148], it is conceivable that other enzymes in addition to cPLA_2_γ may also serve a role as CoA-IT in cells. This is currently an area of active research [130,131,146,147,148,149].

## 7. Lipid Droplets as a Source of Free AA for Lipid Mediator Synthesis

The localization of the eicosanoid-producing enzymes cyclooxygenase and lipoxygenases within LDs as well as the in situ identification of newly formed prostaglandins by immunolabeling techniques, has provided compelling evidence to regard these organelles as specialized intracellular sites for eicosanoid biosynthesis, most often associated with innate immune and inflammatory responses [150,151,152]. Additional evidence linking LD formation with prostaglandin synthesis during inflammation was also provided by a recent study showing that inhibition of TG biosynthesis prevents LD biogenesis and strongly blunts formation of a number of inflammatory mediators including prostaglandin E_2_ [153].

**Figure 5:**
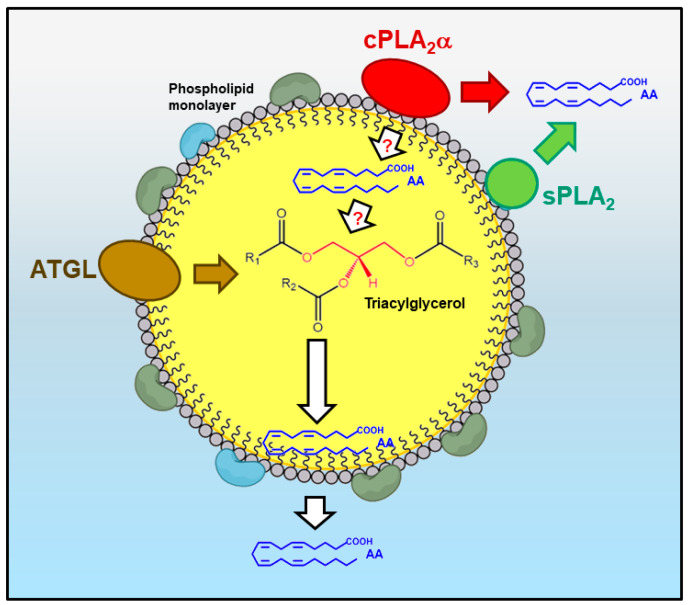
The lipid droplet as a source of AA for lipid mediator production. (i) The direct hydrolysis of the phospholipid monolayer by cPLA_2_α (red) is considered the major pathway. The free AA produced could either be used for eicosanoid synthesis or reacylated into neutral core lipids such as triacylgycerol. (ii) The action of sPLA_2_ (green) on the phospholipid monolayer provides an alternative route for AA mobilization. (iii) Release of AA from the inner core triacylglycerol, a process initiated by ATGL (brown), can provide additional free fatty acid for eicosanoid synthesis or to replenish the fatty acid lost in the phospholipid monolayer by the action of cPLA_2_α and/or sPLA_2_.

The association of the major AA-releasing enzyme cPLA_2_α with the LD monolayer [19,20] also strengthens the link between LDs and eicosanoid synthesis (Figure 5). In this particular aspect, however, it should be kept in mind that the ability of cPLA_2_α to directly hydrolyze AA-containing phospholipids at the LD monolayer has not been demonstrated [154]. Likewise, despite the fact that phospholipid-bound AA has been found in the LD monolayer [151], it has not been demonstrated either that the organelle itself can constitute a significant source of free AA for eicosanoid synthesis. It is possible that the free fatty acid derives from neighboring structures (i.e., ER, Golgi) which the cPLA_2_α also associates with [34] and is transferred to the cyclooxygenases and lipoxygenases residing in the LD by specific transporters.

In addition to a possible role in liberating AA in situ, cPLA_2_α is also well known to play a direct role in regulating the biogenesis of the LD itself [24]. This role implies, among other actions, to promote the accumulation of lysophospholipids at precise locations on the LD monolayer [24]. Formation of lysophospholipids does not necessarily imply that cPLA_2_α is acting upon AA-containing phospholipids, for the enzyme is also capable of hydrolyzing phospholipids without AA if the former are not readily available [128,155]. Moreover, cPLA_2_α roles not involving its hydrolytic activity on AA-containing phospholipids have been described [156,157,158,159]. Given that, as discussed in the preceding section, certain sPLA_2_ enzymes can also act to liberate AA at intracellular locations [133,134,135,136,137,138], it is possible that the LD monolayer also constitutes a site for sPLA_2_ hydrolysis. Serum lipoproteins, which are structurally similar to LDs, are readily hydrolyzed by a variety of sPLA_2_s [160] (Figure 5).

Circulating leukocytes generally contain low levels of AA esterified in TG and CE. However, this situation dramatically changes when the cells become exposed to an exogenous source of free fatty acid, as is the case of monocytes interacting with damaged endothelium [60,75] or leukocytes migrating to lung tissues in response to infection and inflammation [161]. Under these circumstances, the cells rapidly and efficiently take up the AA and incorporate it at large quantities in TG, thus making abundant cytoplasmic LDs. Ultimately, the cells may end up storing AA in TG at levels comparable to those found in membrane phospholipids, sometimes even higher. This process can be reproduced in vitro by incubating the cells with micromolar amounts of free AA [104,161,162], which has allowed a simple, direct means to test whether AA in TG constitutes a mobilizable pool that contributes to lipid mediator synthesis. The studies however failed to detect evidence for a significant role of TG as a supplier of free AA during stimulation conditions, pointing to the AA phospholipid pool as the most relevant contributor to AA release [104,162]. This negative finding gives rise however to the intriguing question of why the cells incorporate such large amounts of AA into a non-mobilizable lipid pool. Given that high levels of available free AA are toxic to cells [103,163], it is possible that sequestration of AA in TG protects the cells from the deleterious effects of the fatty acid. It is possible as well that the incorporation of AA into TG may constitute a buffering mechanism utilized by the cells to limit the amount of fatty acid available for lipid mediator synthesis, providing in this manner a means to modulate the extent and/or duration of pathophysiological responses mediated by AA-derived mediators.

An important unanswered question regarding AA pools in LDs relates to the possible existence of fatty acid exchange mechanisms between phospholipids and TG. If the phospholipid monolayer actually constitutes a significant source of free AA, integrity of the organelle requires the rapid replenishment of the AA that has been freed from the monolayer with another fatty acid. The possibility exists that the TG present in the inner core of the LD may serve this function and supplies the requisite fatty acid [21,154] (Figure 5). Although that such fatty acid recycling mechanism operates in cells and involves AA is yet to be demonstrated, the concept is significant because leukocytes are endowed with all the machinery required to transfer AA from TG to phospholipids via CoA-dependent reactions [164]. Following a similar line of thought, it is challenging to speculate with the possibility that the opposite set of reactions, i.e., AA release from TG which might be replenished by AA coming from the phospholipid monolayer, could also be operative under tightly controlled settings. In partial support of this notion, recent work has implicated ATGL-mediated TG hydrolysis at the LDs in sourcing free AA for lipid mediator production in neutrophils and mast cells [113,114]. Thus, it appears likely that multiple routes governing AA dynamics at the LD exist, and that the contribution of each pathway may vary in a tissue-, cell-, and stimulus-dependent manner [21,165,166].

## 8. Conclusions

For many years, LDs were only thought of as storage organelles for neutral lipids to be mobilized in the case of energy needs. Today we know that, in addition to that passive role, LDs also serve a wide variety of dynamic functions in innate immunity and inflammation. They participate in the development and progression of inflammatory metabolic disorders, of which the most common is cardiovascular disease. In particular, these organelles have been identified as intracellular platforms for lipid signaling enzymes to dock and interact. Although many open questions remain, especially at the level of molecular mechanistic details, it is now clear that LDs play critical roles in the production and regulation of AA-derived mediators in major immunoinflammatory cells. The possible role of TG and CE as sources for the generation of bioactive lipid mediators needs to be further established. Likewise, the importance of these neutral lipids as reservoirs that may restrict the intensity of the innate immune response needs to be considered as well. These studies may uncover new promising targets for therapeutic intervention. Moreover, the discovery that several lipases and phospholipases participate in the generation of lipid mediators from LD provides new challenges and prospects to develop strategies aimed at modulating LD biology. These enzymes may act either in concert or independently of each other, which offers additional opportunities to unveil and explore pathways to treat metabolic disorders. Future studies should also investigate the regulation of LD lipolysis in the context of whole cell signaling and the interactions of LD with other cell compartments which are also known to participate in these processes.

## Data Availability

Not applicable.

## Abbreviations

AA	arachidonic acid
ATGL	triacylglycerol lipase
CE	cholesterol esters
ER	endoplasmic reticulum
LD	lipid droplet
LDL	low density lipoprotein
PA	phosphatidic acid
PC	choline-containing glycerophospholipids
PE	ethanolamine-containing glycerophospholipids
PI	phosphatidylinositol
PS	phosphatidylserine
PUFA	polyunsaturated fatty acid
PLA_2_	phospholipase A_2_
cPLA_2_α	group IVA cytosolic phospholipase A_2_α
cPLA_2_γ	group IVC cytosolic phospholipase A_2_γ
iPLA_2_β	group VIA calcium-independent phospholipase A_2_β
sPLA_2_	secreted phospholipase A_2_
TG	triacylglycerol
